# Enlargement of the brachial plexus on magnetic resonance imaging: a novel finding in adult-onset Krabbe disease

**DOI:** 10.1259/bjrcr.20150213

**Published:** 2016-07-28

**Authors:** Takashi Hiyama, Tomohiko Masumoto, Tadashi Hara, Akira Kunimatsu, Naomi Mamada, Nakamagoe Kiyotaka, Minami Manabu

**Affiliations:** ^1^Department of Radiology, University of Tsukuba Hospital, Tsukuba, Japan; ^2^Department of Radiology, Graduate School of Comprehensive Human Sciences, University of Tsukuba, Tsukuba, Japan; ^3^Department of Radiology, Graduate School of Medicine, University of Tokyo, Tokyo, Japan; ^4^Department of Neurology, Graduate School of Comprehensive Human Science, University of Tsukuba, Tsukuba, Japan; ^5^Department of Neurology, Division of Clinical Medicine, Faculty of Medicine, University of Tsukuba, Tsukuba, Japan

## Abstract

Adult-onset Krabbe disease is an autosomal recessive degenerative leukodystrophy that presents with bilateral corticospinal tract involvement on MRI. Although peripheral nerve involvement is a known manifestation of Krabbe disease, MRI findings of peripheral nerve abnormalities are limited to the cranial nerves and spinal nerve roots. In this case report, we discuss two cases of adult-onset Krabbe disease with brachial plexus enlargement on MRI. Adult-onset Krabbe disease should be included in the differential diagnoses when brachial plexus enlargement and white matter lesions involving corticospinal tracts present simultaneously.

## Summary

Adult-onset Krabbe disease is an autosomal recessive degenerative leukodystrophy that presents with bilateral corticospinal tract involvement on MRI. Although peripheral nerve involvement is a known manifestation of Krabbe disease, MRI findings of peripheral nerve abnormalities are limited to the cranial nerves and spinal nerve roots. In this case report, we discuss two cases of adult-onset Krabbe disease with brachial plexus enlargement on MRI. Adult-onset Krabbe disease should be included in the differential diagnoses when brachial plexus enlargement and white matter lesions involving corticospinal tracts present simultaneously.

## Clinical presentation and investigations

### Case 1

A 22-year-old female was admitted to our hospital with weakness in her left hand. When she was 20 years old, she began experiencing difficulty with moving her right hand while changing her clothes. MRI of the brain showed hyperintensity of the left corticospinal tract on *T*_2_ weighted images. The symptom improved slightly without treatment. When she was 22 years old, she began to feel numbness in both hands and weakness in her left hand. Subsequently, the numbness spread to both upper extremities.

Neurological examinations showed atrophy of both hands and feet, decreased muscle tonus and loss of tendon reflexes of the bilateral upper and lower extremities. Loss of sensation in the right side of the body and face was also noted. Conduction velocity of motor and sensory nerves decreased bilaterally in the upper extremities (motor nerve conduction velocity 25.7 m s^–1^, sensory nerve conduction velocity 19.3 m s^–1^ and compound muscle action potentials 10.8 mV in right median nerve). Galactosylceramidase (GALC) activity in white blood cells decreased to 0.17 nmol mg^–1^ h^–1^ (reference value: 1.75–8.23 nmol mg^–1^ h^–1^), and the patient was diagnosed with adult-onset Krabbe disease. A genetic test for GALC mutation was not performed.

### Case 2

A 50-year-old female who had previously been diagnosed with adult-onset Krabbe disease was admitted. She had experienced bilateral upper and lower extremity weakness since her 30s. Her symptoms had slowly progressed and neurological examinations when she was 40 years old revealed atrophy of muscles in bilateral lower and upper extremities, loss of superficial sensation in the right hand and both feet and a spastic waddling gait. The conduction velocity of the motor and sensory nerves had decreased bilaterally in the upper and lower extremities. *T*_2_ weighted MR images of the brain showed hyperintensities along both corticospinal tracts. The decrease in the white blood cell GALC activity to 0.053 nmol mg^–1 ^ h^–1^ (reference value: 1.93–5.58 nmol mg^–1 ^ h^–1^) was indicative of adult-onset Krabbe disease. A genetic test for GALC mutation was not conducted. When she reached 50 years of age, the weakness had progressed further, and she was admitted to our hospital for rehabilitation.

Neurological examinations showed bilateral atrophy in the upper and lower extremities. Her muscle tonus was flaccid in the upper extremities and spastic in the lower extremities. Bilateral sensory deficits were detected in the upper and lower extremities. A nerve conduction study revealed further decrease in the nerve conduction velocity compared with that recorded previously (compound muscle and sensory nerve action potentials were not detectable in bilateral median nerves).

## Imaging findings

**Figure 1.**
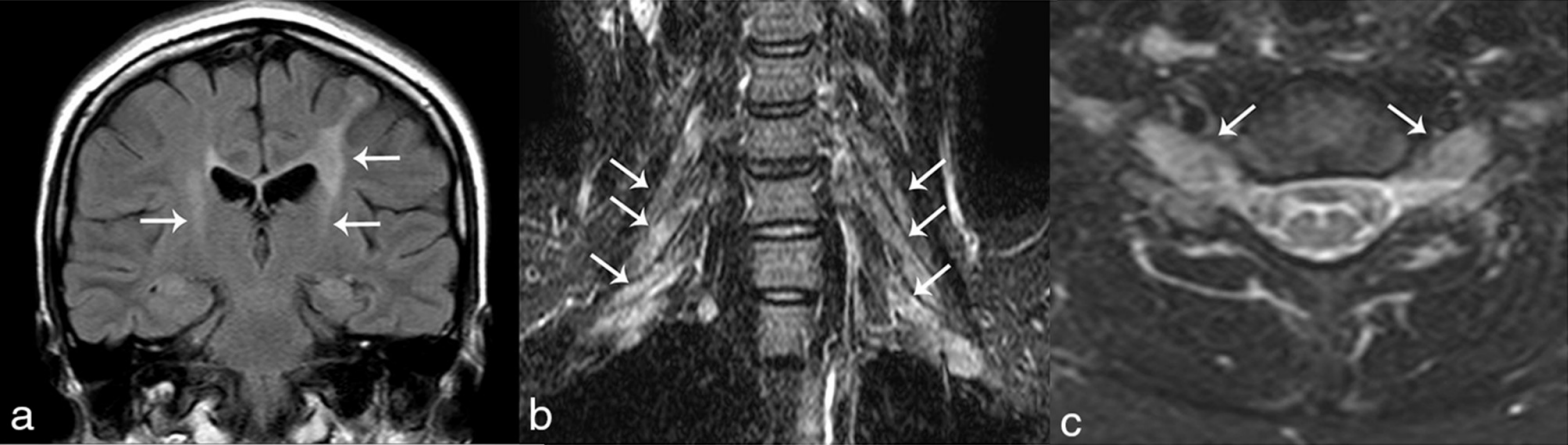
Coronal fluid-attenuated inversion recovery image shows hyperintensities along the bilateral corticospinal tracts (arrows in a). Coronal and axial short tau inversion-recovery images demonstrate enlargement of the bilateral brachial plexus (arrows in b and c).

**Figure 2.**
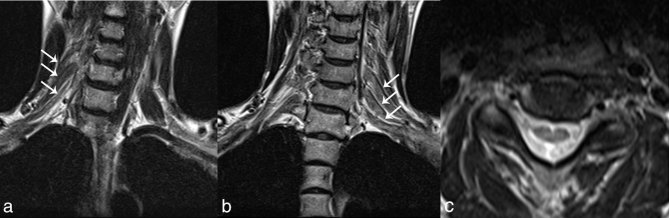
Coronal fast spin-echo *T*_2_ weighted MR images show enlargement of the bilateral brachial plexus (arrows in a and b). Axial fast spin-echo *T*_2_ weighted MR image demonstrates atrophy of the cervical spinal cord (c).

Fluid-attenuated inversion recovery images of the brain demonstrated hyperintensities along bilateral corticospinal tracts in case 1 (Figure 1a). In both cases, MRI of the cervical spine was performed. Short tau inversion-recovery images in case 1 (Figure 1b,c) and *T*_2_ weighted MR images in case 2 (Figure 2a,b) showed bilateral brachial plexus enlargement. Cervical spinal cord atrophy was observed in case 2 (Figure 2c). Spinal cord atrophy was also demonstrated on MRI of the lumbar spine (not shown), but enlargement of the lumbar spinal nerves was not obvious. In case 1, spinal cord atrophy was not observed. Gadolinium-enhanced studies were not conducted in either case.

## Differential diagnosis

Charcot–Marie–Tooth disease and chronic inflammatory demyelinating polyneuropathy were considered in the differential diagnosis for brachial plexus enlargement. However, bilateral corticospinal abnormalities with peripheral neuropathy were indicative of Krabbe disease and metachromatic leukodystrophy. In both cases, the decrease in GALC activity led to the diagnosis of adult-onset Krabbe disease. Brachial plexus enlargement was considered to be caused by Krabbe disease.

## Treatment, outcome and follow-up

Case 1 received supportive treatment only, and the patient’s neurological symptoms were stable during follow-up over the subsequent 2 years. Case 2 received rehabilitation, and the patient was able to live independently with the aid of social services.

## Discussion

Krabbe disease, which is also known as either globoid cell leukodystrophy or galactosylceramide lipidosis, is a rare autosomal recessive demyelinating disorder that is caused by decreased levels of GALC, which induces apoptosis in oligodendrocyte cell lines. Krabbe disease is classified as early and late infantile-, adolescent- and adult-onset types. The infantile-onset type is the most frequent, whereas the adult-onset type is the least frequent. Both cases reviewed here are classified as adult-onset type Krabbe disease.

MRI findings differ among the various types.^1,2^ The adult-onset type tends to show mild cerebral white matter lesions compared with the infantile-onset type. Lesions appear to be relatively limited on both corticospinal tracts. Cerebral atrophy occurs at a later stage for all types and spinal cord atrophy, which was observed in case 2, has also been reported.^3^

Cranial nerve enhancement and enlargement were documented in MR images of the peripheral nerves in Krabbe disease.^4,5^ With regard to spinal nerve root abnormalities, three reports, which included five cases of early and late infantile-onset Krabbe disease, were available.^6–8^ Although these cases showed abnormal enhancement of nerve roots and cauda equina, enlargement of the brachial plexus was not reported. To the best of our knowledge, our present case report will be the first to report brachial plexus enlargement in adult-onset Krabbe disease.

Histopathologically, the peripheral nerves in Krabbe disease showed demyelinating neuropathy characterized by thin myelin sheaths and onion bulb formations.^9^ These findings are not unique to Krabbe disease and have also been observed in acquired and degenerative demyelinating polyneuropathies. Ultrastructural evidence of inclusions in endoneurial macrophages and Schwann cells is necessary for an accurate diagnosis. The peripheral nerves may be enlarged and firm on gross pathological examination, corresponding with brachial plexus enlargement on MR images. Pathologically, optic nerve enlargement is caused by accumulation of globoid cells containing galactosylceramide; it is believed that other forms of cranial nerve enlargement are caused by similar pathology.^5,10^ Although the causes of brachial plexus enlargement were not confirmed in our present case report, the accumulation of galactosylceramide is assumed to be a contributing factor.

In adult patients with diffuse brachial plexus enlargement, Charcot–Marie–Tooth disease, chronic inflammatory demyelinating polyneuropathy, acute inflammatory demyelinating polyneuropathy and lymphoma are considered differential diagnoses; however, there are no reports of simultaneous occurrences of brachial plexus enlargement and corticospinal tract abnormalities in patients with any of these diseases. Therefore, the combination of brachial plexus enlargement and corticospinal hyperintensities on *T*_2_ weighted MR images may be suggestive of adult-onset Krabbe disease.

## Learning points

1. Adult-onset Krabbe disease may present with brachial plexus enlargement on MR images.
2. Adult-onset Krabbe disease should be included in the differential diagnoses if bilateral brachial plexus enlargement and corticospinal tract abnormalities are demonstrated simultaneously on MR images.

## Consent

Written informed consent was obtained from the patients for publication of this case report, including accompanying images.
